# The *Arabidopsis minE* mutation causes new plastid and FtsZ1 localization phenotypes in the leaf epidermis

**DOI:** 10.3389/fpls.2015.00823

**Published:** 2015-10-06

**Authors:** Makoto T. Fujiwara, Kei H. Kojo, Yusuke Kazama, Shun Sasaki, Tomoko Abe, Ryuuichi D. Itoh

**Affiliations:** ^1^RIKEN Nishina CenterSaitama, Japan; ^2^Graduate School of Science and Technology, Sophia UniversityTokyo, Japan; ^3^Department of Integrated Biosciences, Graduate School of Frontier Sciences, The University of TokyoKashiwa, Japan; ^4^LPixel Inc.Tokyo, Japan; ^5^Department of Chemistry, Biology and Marine Science, Faculty of Science, University of the RyukyusOkinawa, Japan

**Keywords:** *Arabidopsis thaliana*, chloroplast, fluorescent protein, FtsZ, leaf epidermis, MinE, plastid division, stromule

## Abstract

Plastids in the leaf epidermal cells of plants are regarded as immature chloroplasts that, like mesophyll chloroplasts, undergo binary fission. While mesophyll chloroplasts have generally been used to study plastid division, recent studies have suggested the presence of tissue- or plastid type-dependent regulation of plastid division. Here, we report the detailed morphology of plastids and their stromules, and the intraplastidic localization of the chloroplast division-related protein AtFtsZ1-1, in the leaf epidermis of an *Arabidopsis* mutant that harbors a mutation in the chloroplast division site determinant gene *AtMinE1*. In *atminE1*, the size and shape of epidermal plastids varied widely, which contrasts with the plastid phenotype observed in *atminE1* mesophyll cells. In particular, *atminE1* epidermal plastids occasionally displayed grape-like morphology, a novel phenotype induced by a plastid division mutation. Observation of an *atminE1* transgenic line harboring an *AtMinE1* promoter::*AtMinE1-yellow fluorescent protein* fusion gene confirmed the expression and plastidic localization of AtMinE1 in the leaf epidermis. Further examination revealed that constriction of plastids and stromules mediated by the FtsZ1 ring contributed to the plastid pleomorphism in the *atminE1* epidermis. These results illustrate that a single plastid division mutation can have dramatic consequences for epidermal plastid morphology, thereby implying that plastid division and morphogenesis are differentially regulated in epidermal and mesophyll plastids.

## Introduction

**FIGURE 1:**
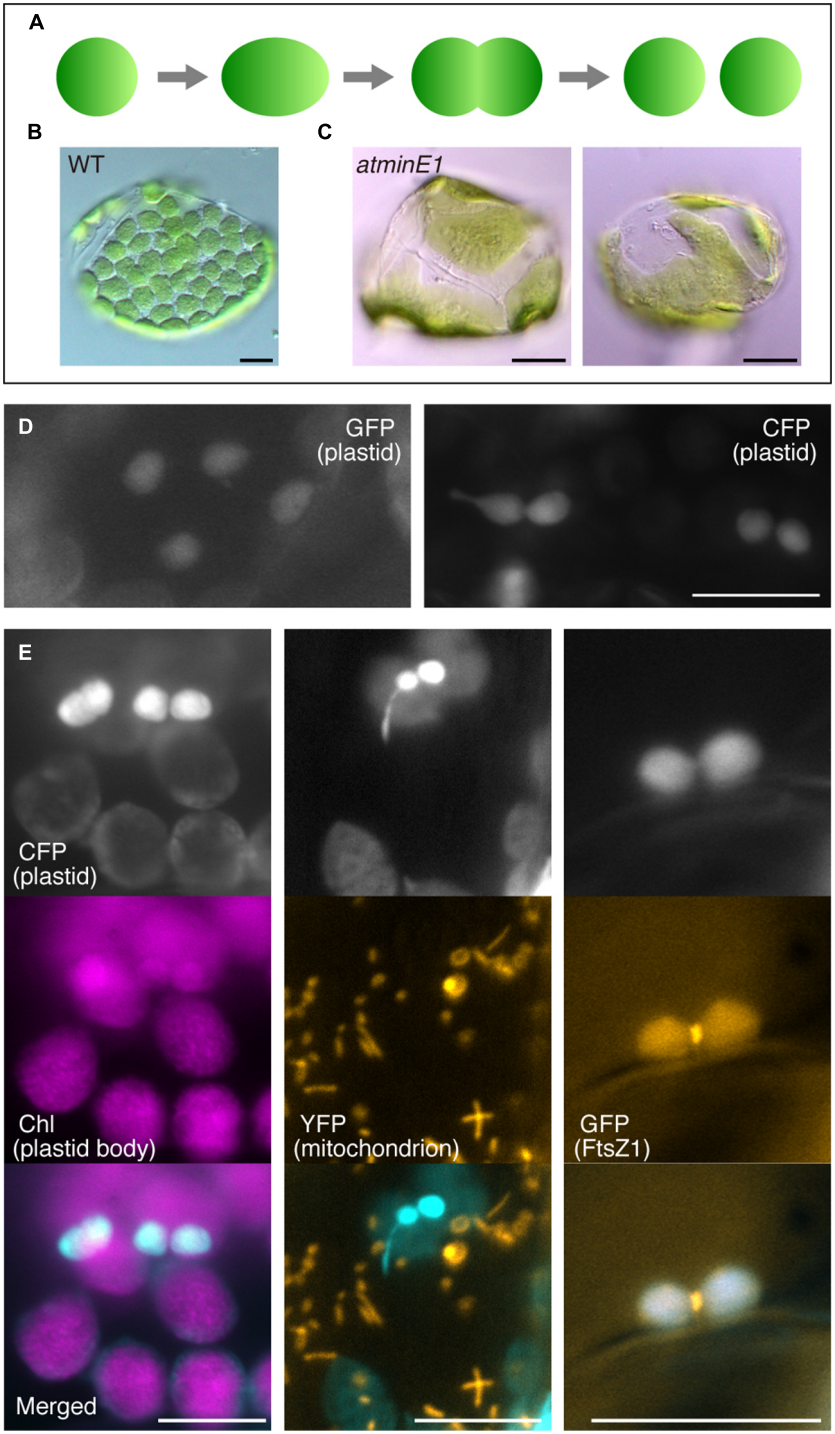
**Utility of cyan fluorescent protein (CFP) to investigate plastid morphology in *Arabidopsis* leaf epidermis. (A–C)** A framework describing the replication and morphology of leaf mesophyll chloroplasts. Schematic diagram of chloroplast replication by binary fission **(A)** and chloroplast phenotypes in WT **(B)** and *atminE1* **(C)** leaf mesophyll cells are shown. **(D)** Detection of plastid-targeted green fluorescent protein (GFP, left) and CFP (right) in leaf epidermis. **(E)** Dual detection of plastid-targeted CFP and chlorophyll (Chl magenta-colored), mitochondria-targeted YFP (orange-colored) or FtsZ1–GFP (orange-colored) in leaf epidermis. **(D,E)** Leaves from 2-week-old seedlings of WT-background transgenic lines were observed by fluorescence microscopy. In merged images, CFP fluorescence is colored in cyan. Bars = 5 μm (black) and 10 μm (white).

Plastids are ancient prokaryote-derived organelles in plant cells that multiply via division of pre-existing organelles. The photosynthetic plastids, chloroplasts, undergo symmetric division, which produces a homogeneous population of spherical chloroplasts in each mature-leaf cell (Possingham and Lawrence, 1983; López-Juez and Pyke, 2005; **Figures 1A,B**). Chloroplast fissions are driven by a macromolecular complex located on the double-envelope membrane at the plastid constriction site. In the model plant *Arabidopsis thaliana*, approximately 10 nuclear-encoded proteins are located at distinct subplastidic compartments, which constitute the division apparatus (Yang et al., 2008; Okazaki et al., 2010). Among these proteins, the prokaryotic tubulin-like protein FtsZ, which stands for “Filamenting temperature-sensitive Z”, plays a central role in the initiation and progress of chloroplast division. *A. thaliana* has two phylogenetically distinct, functionally non-redundant FtsZ proteins, FtsZ1 and FtsZ2 (Stokes and Osteryoung, 2003; Miyagishima et al., 2004), both of which have polymerization and filament-bundling activities, enabling them to form a contractile ring structure at the mid-chloroplast site (McAndrew et al., 2001; Vitha et al., 2001; Yoder et al., 2007).

The symmetry and the “one division site (or one FtsZ ring) at a time” manner of normal chloroplast division in leaf mesophyll cells (**Figure 1A**) rely on at least four chloroplast proteins; MinD (Colletti et al., 2000; Kanamaru et al., 2000), MinE (Itoh et al., 2001; Maple et al., 2002; Reddy et al., 2002), ARC3 (Shimada et al., 2004; Maple et al., 2007), and MCD1 (Nakanishi et al., 2009). MinD and MinE are endosymbiont-derived stromal proteins, while ARC3 is a plant-specific stromal protein harboring an FtsZ-like domain. MCD1 is a plant-specific membrane protein that spans the inner envelope and recruits MinD to the division site. In *A. thaliana*, only one homolog of *minE*, designated *AtMinE1*, is encoded by the nuclear genome (Itoh et al., 2001). Overexpression, repression, or insertional mutation of *AtMinE1* results in the generation of enlarged chloroplasts and a reduced number of chloroplasts per mesophyll cell, although the chloroplast morphology differs among these three plant types (Itoh and Yoshida, 2001; Itoh et al., 2001; Fujiwara et al., 2008). The relationship between *minE* expression levels and the chloroplast morphology phenotype in *A. thaliana* is analogous to that in *Escherichia coli* cells: in both cases, *minE* overexpression results in a heterogeneous population of chloroplasts or cells, whereas its repression generates giant chloroplasts or elongated cells due to inhibited division (de Boer et al., 1989).

The best-characterized pathway of plastid division in plants is chloroplast division in leaf mesophyll cells (Pyke, 1997). The discovery and detailed characterization of a series of *A. thaliana* mutants, the *accumulation and replication of chloroplasts* (*arc*) mutants (Pyke, 1997), bearing a numerically and morphologically unusual population of mature-leaf mesophyll chloroplasts, has revealed a good correlation between the state of chloroplast division apparatus and the mutant chloroplast phenotypes. Namely, the degree of division apparatus disorganization is reflected by the degree of reduction in chloroplast number, as well as the extent of organelle enlargement. In *A. thaliana arc6*, the most severe mutant of chloroplast division, only one or a few giant chloroplasts are generated per leaf mesophyll cell (Pyke et al., 1994) because the FtsZ ring assembly is completely blocked in this mutant (Vitha et al., 2003), although the total chloroplast compartment volume per cell remains constant via a compensatory mechanism (Pyke, 1997). This cause-and-effect relationship fits well with the principle of cell division in organisms. Thus, the chloroplast replication state in terminally differentiated leaf mesophyll cells has been used as a diagnostic aid to determine the significance of target genes in chloroplast division.

In recent years, however, it has become clear that the framework of “division inhibition leading to enlargement of plastids” (**Figure 1C**, for example) is not necessarily applicable to every cell lineage or plastid type. Forth and Pyke (2006) found that the tomato *suffulta* mutation results in the formation of a few giant chloroplasts per cell, but these convert into a wild-type-like population of chromoplasts by budding and fragmentation of plastids or stromules during fruit ripening. Holzinger et al. (2008) examined the effects of *A. thaliana arc3, arc5*, and *arc6* mutations on plastid number and morphology in many plant organs or tissues, showing that stromules are more abundant in several epidermal tissues in these mutants compared to wild type. In addition, Chen et al. (2009) observed plastids in the embryos of *A. thaliana crumpled leaf* (*crl*) and *arc6* mutants, implying that plastid protrusions lacking chlorophylls can serve as precursors of new daughter plastids during cell division. Furthermore, we previously demonstrated that stromule length and frequency increase without a dramatic change in plastid size or number in several tissues of the chloroplast division mutant *atminE1* (Kojo et al., 2009), which harbors a T-DNA insertion in the *AtMinE1* locus and hence produces severely reduced levels of *AtMinE1* transcripts (Fujiwara et al., 2008). These findings prompted us to formulate an alternative framework to explain the relationship between FtsZ-based plastid division and plastid morphogenesis, especially stromule formation, in non-mesophyll cells.

We therefore focused our attention on the leaf epidermis. To date, plastid morphology and division in the leaf epidermis of chloroplast division mutants has largely been overlooked, with the exception of the tomato *suffulta* mutant (Forth and Pyke, 2006), the *A. thaliana msl2 msl3* double mutant (Haswell and Meyerowitz, 2006), and the *atminE1* mutant (Fujiwara et al., 2009). In a previous study (Fujiwara et al., 2009), we observed two distinct types of plastid morphology and an altered configuration of AtFtsZ1-1 (*A. thaliana* FtsZ1) in the leaf epidermis of *atminE1*. Specifically, we observed giant plastids containing short filaments and multiple dots of FtsZ1 and relatively poor plastids containing multiple FtsZ1 rings or spiral(s). The latter type was observed in the leaf epidermis but not in mesophyll cells, suggesting that the plastid division system operates in a tissue-specific manner. Hence, the *atminE1* mutant is well suited for investigating the tissue-specific characteristics of plastid morphology and division. To effectively visualize epidermal plastids (immature chloroplasts), which are only weakly pigmented, we utilized plastid (stroma)-targeted cyan fluorescent protein (CFP). CFP is an effective fluorophore for labeling organelles in the leaf epidermis, because the background fluorescence signals from chloroplasts in the underlying mesophyll layer are relatively low compared to those obtained using green fluorescent protein (GFP; Kato et al., 2002). In fact, the fluorescent images of CFP-labeled plastids in the leaf epidermis were relatively clear and showed a high signal-to-background ratio compared to those from GFP-labeled plastids (**Figure 1D**). In the present study, we employed transgenic *atminE1* lines that stably express a transgene encoding plastid-targeted CFP. Extending our earlier observations (Fujiwara et al., 2009), we explored the detailed morphology of leaf epidermal plastids, stromules, and other types of plastid substructures, the localization of FtsZ1 within the plastids, and their possible associations with plastid constriction in the *atminE1* mutant.

## Materials and Methods

### Plant Materials and Growth Conditions

*Arabidopsis thaliana* (L.) Heynh. ecotypes Columbia (Col) and Wassilewskija (Ws) were used as wild-type (WT) plants. A T-DNA insertional mutant of *AtMinE1*, Flag_056G07 (DLFTV7T3, Ws background), was obtained from Institut National de la Recherché Agronomique (INRA, Versailles, France) (Samson et al., 2002). A transgenic line, Z1g11 (Col background), expressing a full-length AtFtsZ1-1–sGFP(S65T) fusion (FtsZ1–GFP) under an upstream genomic sequence of *AtFtsZ1-1*, and its cross with *atminE1* were previously described (Fujiwara et al., 2008, 2009). Another transgenic line, FL4-4 (Col background), expressing stroma-targeted CFP (TP_FtsZ1-1_–CFP), and matrix-targeted yellow fluorescent protein (YFP; Pre_mtHSP60_-YFP) under the control of the CaMV35S promoter, and its cross with *atminE1*, were also generated previously (Itoh et al., 2010). A stable line, ptA5-3 (Col background), expressing stroma-targeted GFP (TP_RBCS3A_-GFP) under the control of the CaMV35S promoter (Niwa et al., 1999), was provided by Dr. Yasuo Niwa. Plants were germinated and grown as previously described (Fujiwara et al., 2009), except for the duration of cold treatment of seeds being 4 days.

### Generation of Transgenic Line Expressing Stroma-targeted CFP

To monitor plastids in living tissues of *A. thaliana*, a gene cassette expressing an N-terminal transit peptide sequence (90 aa) of AtFtsZ1-1 fused to the N-terminus of CFP (provided by Dr. Atsushi Miyawaki) (TP_FtsZ1-1_–CFP) under the control of the CaMV *35S* promoter and the *NOS* terminator was inserted into the *Hin*dIII and *Eco*RI sites of the binary vector pSMAB704 (Igasaki et al., 2002; provided by Dr. Hiroaki Ichikawa) by simultaneously removing the vector-derived CaMV35S promoter, *uidA*, and *NOS* terminator cassette (see Supplementary Figure S1). The resulting vector, pSMAB-Z1TP-sC, was employed for *Agrobacterium*-mediated Col transformation by the floral dip method (Clough and Bent, 1998). A total of 429 transformed (T_1_) seedlings were selected on bialaphos (4 μg/l, Meiji Seika, Tokyo, Japan)-containing MS plates. Of 13 lines that showed high and stable CFP fluorescence, one line, FC1-7, was chosen for its fluorescence stability over three generations without occurrence of transgene silencing, with the aid of stereofluorescence microscopy (model FLIII; Leica Microsystems, Heidelberg, Germany). FC1-7 was crossed with Z1g11 and Z1g11 × *atminE1* to efficiently visualize the stroma in these lines. The F_3_ progenies were characterized by fluorescence microscopy.

### Complementation Assay of *atminE1* with *AtMinE1-YFP*

A genomic copy of *AtMinE1* was amplified by PCR with oligonucleotide primers E1-9 (5′-GA*G TCG ACC CGG G*TT ACG AAG AAG CCT TGG TTC-3′) and E1-8 (5′-T*GT CGA C*CT CTG GAA CAT AAA AAT CGA ACC-3′) (*Sal*I and *Sma*I restriction sites italicized). The PCR product (2.7 kb), comprising a 1.2 kb upstream genomic region of *AtMinE1* and the *AtMinE1* open reading frame, was introduced into pSMAB704 by simultaneously removing the CaMV35S promoter, *uidA*, and the *NOS* terminator cassette and co-introducing a 1.0 kb *Sal*I-*Eco*RI fragment of the *YFP*::*NOS* terminator cassette (the original *YFP* [*Venus*] was provided by Drs. Takeharu Nagai and A. Miyawaki). The resulting plasmid, pSMAB-E1-V, was employed for *Agrobacterium*-mediated transformation of the *A. thaliana atminE1* mutant (Clough and Bent, 1998). A total of 24 T_1_ seedlings were selected as described above. The T_2_ or T_3_ progenies were analyzed by quantitative RT-PCR, immunoblotting, stereofluorescence microscopy, and epifluorescence microscopy.

### Quantitative RT-PCR Analysis

Total RNA from primary leaves of 2-week-old seedlings of the Ws, *atminE1*, and transgenic *atminE1* lines (overexpression line [E1v10], complemented line [E1v24]) was extracted and subjected to quantitative RT-PCR as previously described (Fujiwara et al., 2010). The primers used were as follows: for *18S rRNA* (used as an internal control), 18SrRNAF6: 5′-GAC TAC GTC CCT GCC CTT TGT-3′ and 18SrRNAR6: 5′-ACT TCA CCG GAT CAT TCA ATC G-3′; for *AtMinE1*, AtMinE1-FOR4: 5′-TCA TTA CCT TCT TCT TCT TCC-3′ and AtMinE1-REV4: 5′-TGC AAG AAC CTT CAC CTG ACC-3′; and for *AtMinD1*, MD-FOR3: 5′-AAT GGC GAC AAC TGA GAA ACC-3′ and MD-REV: 5′-CGC GTA TCG TCG TTA TCA CCT-3′.

### Western Blotting

Total proteins were extracted from aerial parts (∼50 mg) of 17-day-old soil-grown *A. thaliana* plants and subjected to Western blotting as previously described (Fujiwara et al., 2009). The band intensity was quantified with image processing software ImageJ version 1.43j (http://rsb.info.nih.gov/ij/).

### Stereofluorescence Microscopy

Whole seedlings or floral organs were observed under a stereofluorescence microscope (FLIII [Leica Microsystems]) equipped with a CCD digital camera (ORCA-ER [Hamamatsu Photonics, Hamamatsu, Japan]). YFP and chlorophyll signals were detected with standard filter sets for EYFP (Leica; excitation: 500–520 nm; emission: 540–580 nm) and Texas Red (excitation: 540–580 nm; emission:>610 nm), respectively. Digital black-and-white images were processed using Adobe Photoshop (Adobe Systems Inc., San Jose, CA, USA).

### Epifluorescence Microscopy

Whole plant organs were mounted under glass coverslips and observed under an epifluorescence microscope (IX70 [Olympus, Tokyo, Japan], equipped with ORCA-ER [Hamamatsu Photonics]) using 60× (numerical aperture [N.A.] 1.20, water immersion), 60× (N.A. 1.35, oil immersion), and 100× (N.A. 1.40, oil immersion) objective lenses (Olympus). Stroma-targeted GFP was detected with a filter cube U-MWIBA (Olympus; excitation: 460–490 nm; emission: 510–550 nm). FtsZ1–GFP and chlorophyll autofluorescence were detected as described previously (Fujiwara et al., 2009). CFP was detected with CFP-2432A (Semrock, Rochester, NY, USA; excitation 426–450 nm; emission 465–501 nm). To avoid rapid photobleaching of fluorescent proteins and to minimize photoresponses of plant cells, samples were observed at 6–25% excitation strength. No chloroplast photorelocations were observed during microscopy. Digital black-and-white images were imported into RGB channels of Adobe Photoshop CS3 to obtain the final merged images. To obtain line profile data, original images of CFP and GFP were aligned with ImageJ plugin StackReg, available at http://bigwww.epfl.ch/thevenaz/stackreg/. Noise reduction was performed by band-pass filtering using KBI plugins.

## Results and Discussion

### Plastid Morphology and Division in the Leaf Epidermis of Wild-type Plants

Leaf epidermal plastids are relatively small and underdeveloped (as suggested by the weak autofluorescence from chlorophyll) compared with mesophyll chloroplasts (**Figure 1E**, left). These plastids are often present in dumbbell- or peanut-shaped form. The number, shape, size, and intracellular distribution of the leaf epidermal plastids in WT observed in the present study were basically in accordance with previous reports (Chen et al., 2009; Schattat and Klösgen, 2011). Dual detection of stroma-targeted CFP and FtsZ1–GFP [AtFtsZ1-1–sGFP(S65T)] (Fujiwara et al., 2008) in a transgenic line (see Materials and Methods for details) revealed that the peanut-shaped plastids in the leaf epidermis were associated with the production of centrally located FtsZ1–GFP signals within the plastids (**Figure 1E**, right). This result indicates that these plastids were in the process of FtsZ1 ring-mediated symmetric division, in a similar manner to mesophyll chloroplast division. Nevertheless, epidermal plastids displayed a greater tendency to form stromules and thus a higher plasticity in envelope morphology than mesophyll chloroplasts.

While a detailed model of mesophyll chloroplast division has been proposed (Gao and Gao, 2011; Basak and Møller, 2013; Osteryoung and Pyke, 2014), which is mainly based on thorough observations of mutants and transgenic lines with altered chloroplast size and number, no such model has been established for non-mesophyll plastids, as morphological studies of each type of plastid have not previously been performed. To gain insight into epidermal plastid division, we conducted intensive observations of plastid morphology in the leaf epidermis of the *A. thaliana atminE1* mutant, one of the most severe mutants of chloroplast division (**Figure 1C**; Fujiwara et al., 2008). The *atminE1* mutant harbors a T-DNA insertional mutation at intron 1 of the *AtMinE1* locus, resulting in approximately 2,000-fold reduction in the *AtMinE1* transcripts as compared to those in WT (Fujiwara et al., 2008).

### Detailed Morphology of Leaf Epidermal Plastids in the *atminE1* Mutant

**FIGURE 2:**
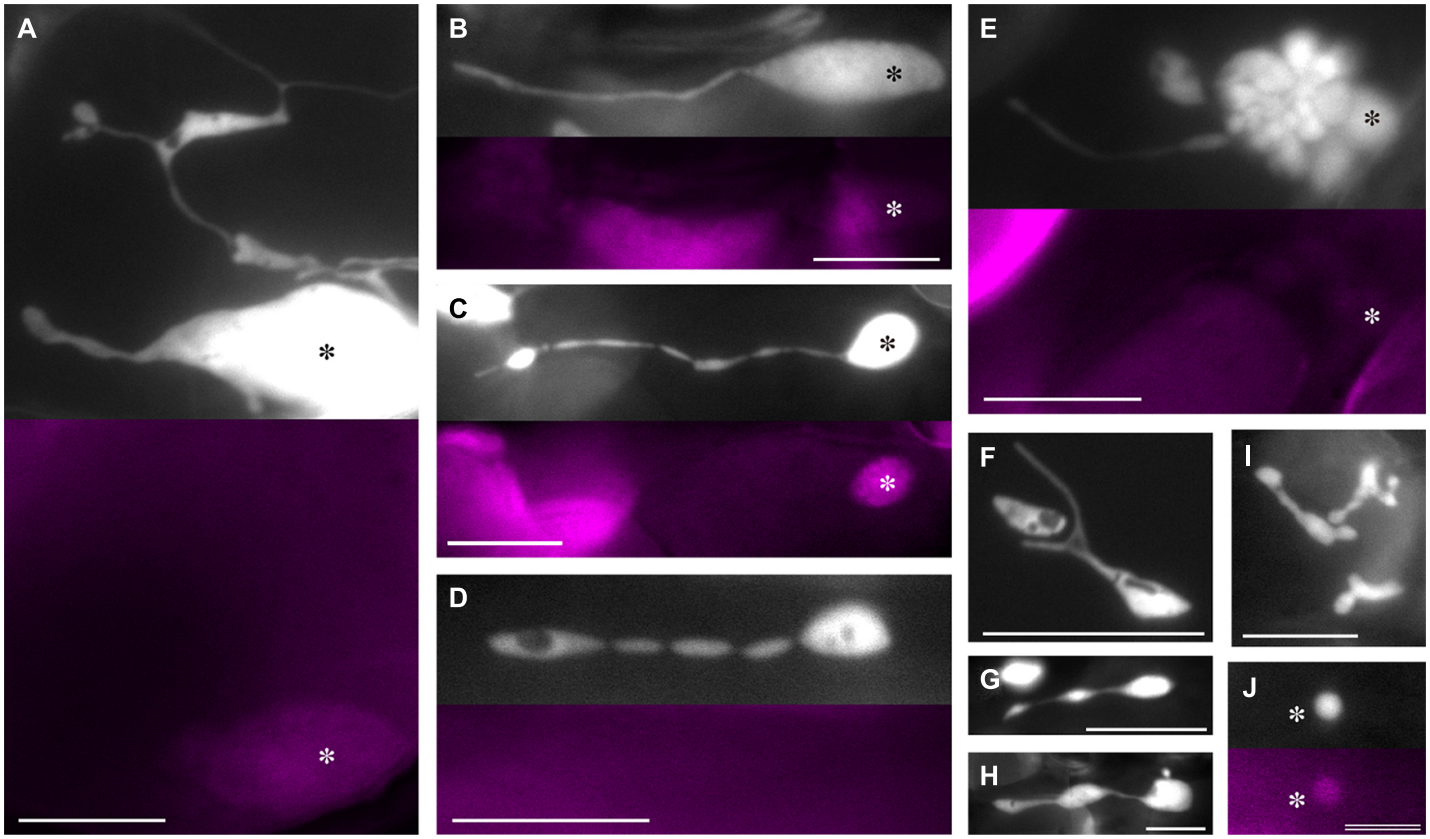
**Plastid morphology in leaf epidermis of *atminE1*. (A–J)** Images of CFP-labeled plastids in leaf petiole epidermis of 2- or 3-week-old *atminE1* seedlings. Fluorescence images of chlorophyll (colored in magenta) are also shown. Asterisks indicate plastid bodies with positive chlorophyll signals. Plastids in **(D,F–I)** lack signals. Bars = 10 μm **(A–I)** and 5 μm **(J)**.

Taking advantage of the merits of plastid-targeted CFP described above, we investigated the plastid morphology in leaf petiole epidermis of 2- and 3-week-old *atminE1* seedlings using epifluorescence microscopy (**Figure 2**). While epidermal (pavement) cells of the leaf blade are puzzle piece-shaped with interdigitated lobes, those of the petiole assume a flat rectangular shape, making them more suitable for the observation of plastids and other organelles. First, we focused on the morphology of individual plastids. Even in the same cell, plastids were highly polymorphic in terms of their subplastidic structures such as main plastid bodies, stromules, and bulges. Importantly, it was often difficult to distinguish among these three structures (**Figure 2A**), which is often the case for non-green plastids (Schattat et al., 2015). Nonetheless, we typically observed giant plastids with long stromules (**Figure 2B**). As variations of this typical form, we observed shallow, wavy constrictions on stromules (**Figure 2C**) and seemingly fragmenting stromules (**Figure 2D**). These stromules often contained substructures resembling plastid bodies in their interior or terminal regions (**Figures 2C,D**). These characteristic morphologies of stromules were observed regardless of the presence of chlorophyll autofluorescence in their main plastid bodies. The *atminE1* mutant also exhibited a unique morphological feature in the plastid bodies of the leaf epidermis. At low frequency, mini-sized plastid bodies aggregated into a grape-like clump (**Figure 2E**). In some cases, some of the plastid bodies within a clump emitted faint chlorophyll autofluorescence. Also, we noticed the presence of relatively immature, chlorophyll-free plastids, like those observed in the leaf epidermis of the *A. thaliana arc6* mutant (Holzinger et al., 2008). This type of plastid assumed various shapes, appearing round, stretched, or with multiple constrictions (**Figures 2F–I**). We did not detect a significant relationship between plastid size and shape (**Figures 2G,H**), except for the predominance of a round form among small, poorly developed plastids. Another phenotype that is unique to the *atminE1* epidermis among chloroplast division mutants examined thus far is the presence of mini-sized, chlorophyll-containing plastids (**Figure 2J**). Tiny plastids in the epidermis of chloroplast division mutants, which were previously reported, lacked chlorophyll (Holzinger et al., 2008).

In summary, we detected novel, unique phenotypes of leaf epidermal plastids in *atminE1*, which have not been reported in other *A. thaliana* mutants that exhibit severely impaired chloroplast division. These phenotypes include the formation of grape-like plastid clusters (**Figure 2E**) and tiny plastids with positive chlorophyll autofluorescence signals (**Figure 2J**), while *atminE1* epidermal plastids also exhibited enlarged plastid bodies and excessive stromule formation (**Figures 2A–D**), both of which are common phenotypes of non-green plastids in severe chloroplast division mutants. These results imply that the latter “common” phenotypes reflect the fundamental effects of inhibited division whereas the former “unique” phenotypes represent an additional effect that depends on the specific function of AtMinE1 or the severity of inhibited division.

### Size, Number, and Intracellular Distribution of Leaf Epidermal Plastids in the *atminE1* Mutant

**FIGURE 3:**
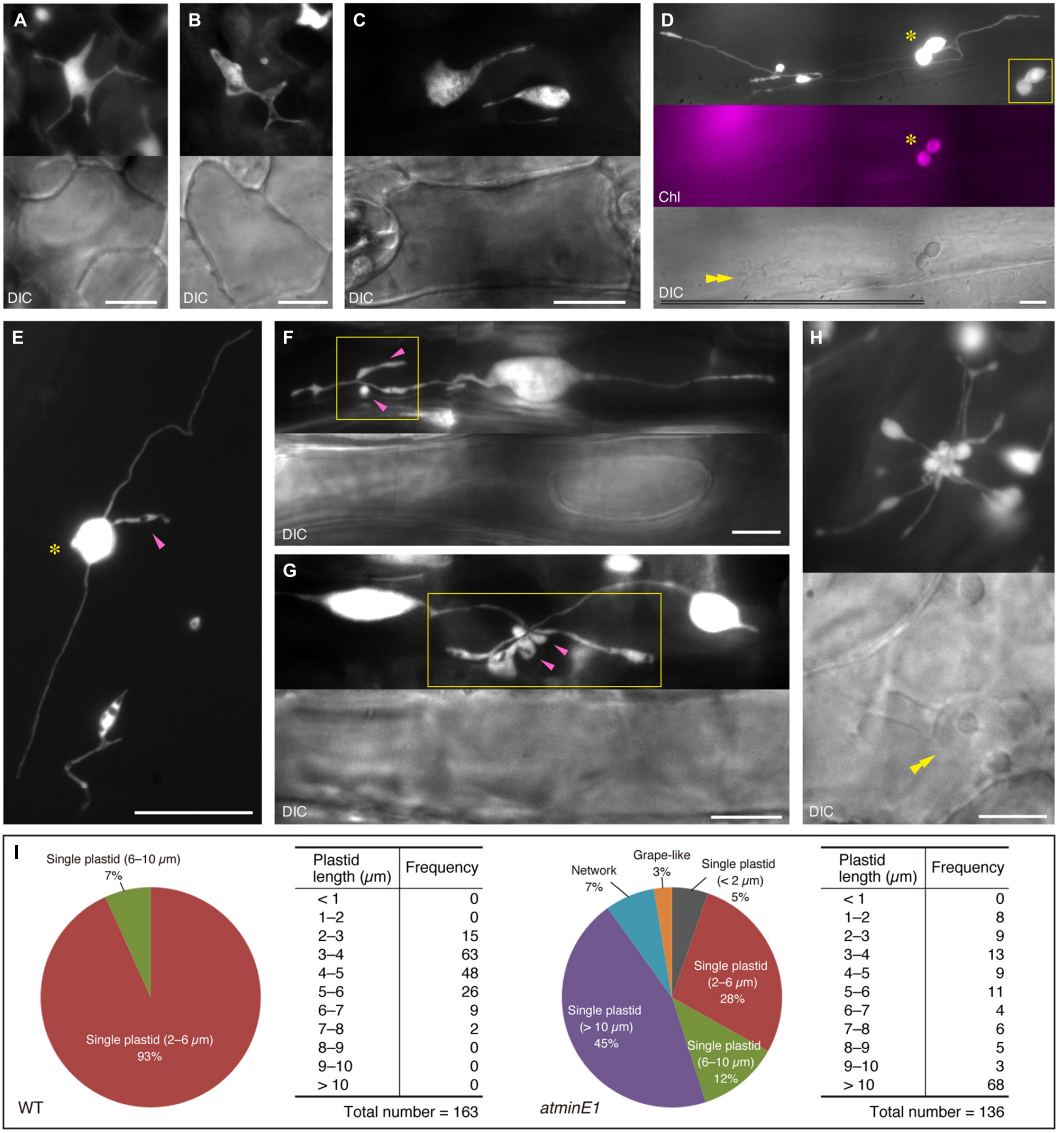
**Various types of plastid morphology and distribution patterns in leaf epidermis of *atminE1*. (A–H)** Images of CFP-labeled plastids in leaf petiole epidermis of 2- or 3-week-old *atminE1* seedlings. DIC and chlorophyll fluorescence (Chl magenta-colored) images are also shown. Asterisks indicate plastids with chlorophyll signals only within cells, while double arrowheads indicate cell nuclei. Single arrowheads and boxes represent plastid bulges associated with plastid bodies or stromules and their activated regions, respectively. Inset in **(D)** is a CFP image of a chlorophyll-positive plastid pair taken using a shorter exposure time. Bars = 10 μm (white) and 100 μm (black). **(I)** Measurement of plastid morphologies in WT and *atminE1*. Phenotypes of epidermal plastids in 2-week-old seedlings were classified into three groups, ‘single plastid’, ‘network’ and ‘grape-like’. The former group was further examined with respect to total plastid length. Plastid length was defined as the length of the longest line passing over the plastid area. Since the borders between plastid bodies and stromules were often unclear in *atminE1*, the plastid length includes the area of stromules (for both WT and *atminE1* samples).

Next, we examined the plastid morphology in the leaf petiole epidermis of *atminE1* in light of their size, number, and distribution within each cell (**Figure 3**). We found several plastid size, number, and distribution patterns: a single cell could contain only one giant plastid (**Figure 3A**), one giant plastid coexisting with tiny plastid(s) (**Figure 3B**), or some plastid bodies connected by stromules, forming a network throughout the entire length of the petiole epidermal cell (**Figure 3C**; Supplementary Figure S2A). These patterns are basically in agreement with the findings of a previous report on the *arc6* mutant (Holzinger et al., 2008). Moreover, we occasionally detected a cell containing dumbbell-shaped, chlorophyll-bearing plastids (**Figure 3D**), a novel feature of *atminE1*. In general, only giant plastids, which appeared to be produced via inhibited plastid division, appeared to contain chlorophyll (**Figures 2A–C**) in the *atminE1* epidermis, although there were some exceptions (**Figures 2E,J**). The existence of dumbbell-shaped, chlorophyll-bearing plastids implies that some of these chlorophyll-containing plastids maintain the capability of proliferation by division.

While exploring how and why the heterogeneity of plastid shape and size in each individual cell was generated in *atminE1*, we noticed bulge- or vesicle-like subplastidic structures attached to the main plastid bodies or stromules (**Figure 3E**, magenta arrowhead, and Supplementary Figure S2B). These structures usually underwent transformation, even during short observation periods, but they remained firmly attached to other parts of the plastids (i.e., bodies and stromules). We frequently observed that the generation of such bulge-like structures was strongly activated in a confined region of plastids (**Figures 3F,G**, boxes). In all images shown in **Figures 3E–G**, all of the bulge- or vesicle-like structures and their accompanying plastid bodies or stromules were located in the same focal plane. Notably, those bulges appeared to emerge from a single point, although no discrete structure (such as a “central node”) was identified. These observations suggest that the grape-like plastid clusters (**Figures 2E** and **3H**, Supplementary Figure S2C) represent an extreme state resulting from strong activation of bulge and/or vesicle formation. **Figure 3H** shows an example of a plastid cluster juxtaposed with the cell nucleus. Indeed, plastid bodies, stromules, and bulges were often located near nuclei in the leaf epidermal cells of *atminE1*.

Taken together, these findings demonstrate that the leaf epidermis of *atminE1* is characterized by a heterogeneous cell population containing various types (**Figure 3I**) of plastid morphology and distribution patterns.

### Examining the Relationship between Leaf Epidermal Plastids and Mitochondria in the *atminE1* Mutant

The findings described above demonstrate that leaf epidermal plastids in *atminE1* could assume various shapes. A sub population of the plastids and their substructures closely resembled mitochondria. Indeed, the size and shape of stromules and mitochondria, their resemblance to each other, and even the potential relationships between these structures have been discussed previously (Wildman et al., 1962; Kwok and Hanson, 2004). To investigate the possible relationship between subplastidic structures (bulges and stromules) and mitochondria, we employed a transgenic *A. thaliana* line, FL4-4, which stably expresses both mitochondrion-targeted YFP and plastid-targeted CFP, as well as *atminE1* harboring both marker genes (transferred from FL4-4 by crossing; Itoh et al., 2010). Using these lines, we conducted simultaneous detection and morphological comparisons of both organelles.

**FIGURE 4:**
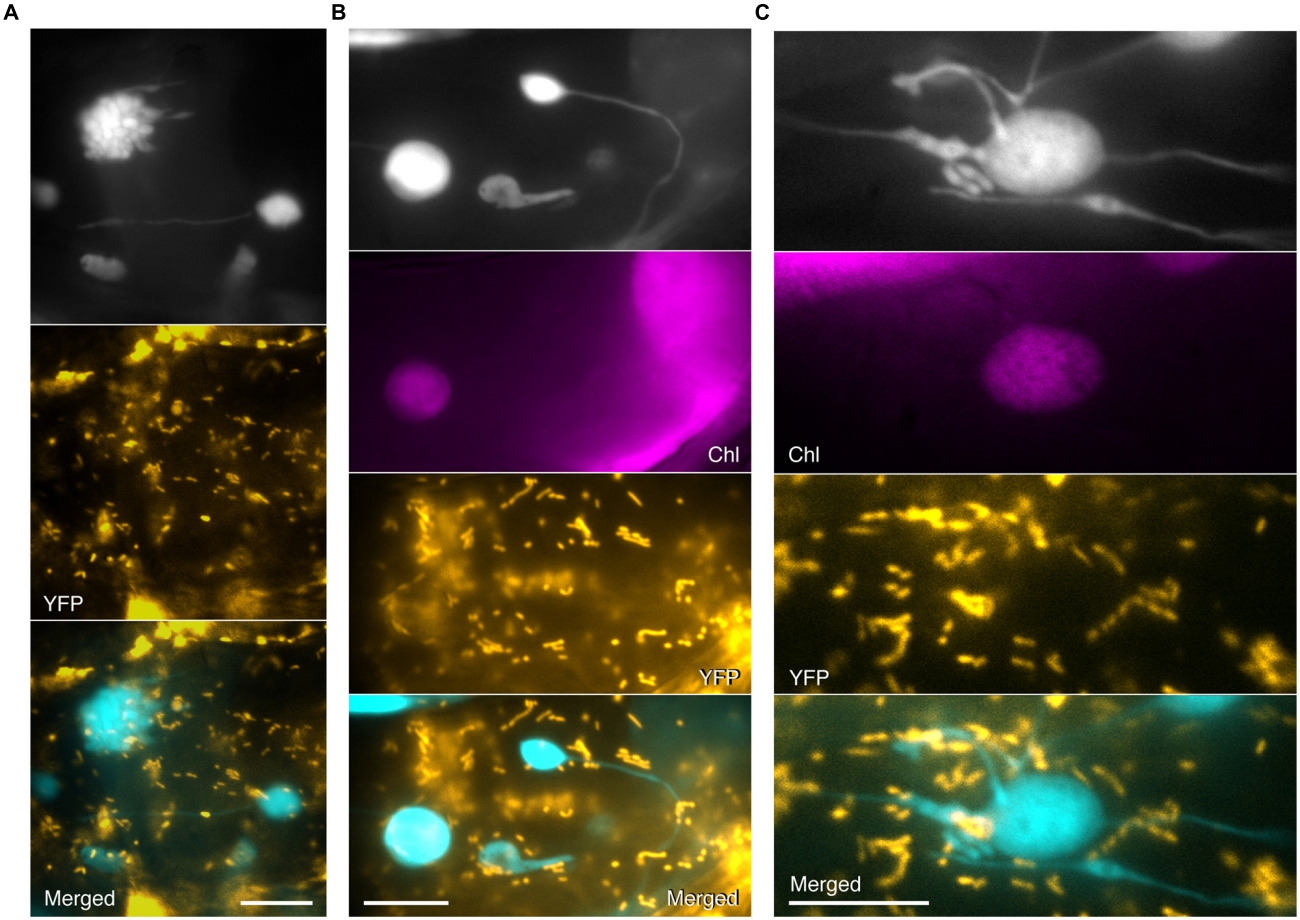
**Simultaneous detection of plastids and mitochondria in leaf epidermis of *atminE1*. (A–C)** Images of plastids and mitochondria in leaf petiole epidermis of 2- or 3-week-old *atminE1* seedlings. Images of plastid-targeted CFP (top in black and white), chlorophyll (Chl magenta-colored), mitochondria-targeted YFP (orange-colored), and merged (CFP cyan-colored, YFP orange-colored) are shown. Bars = 10 μm.

No colocalization of CFP signals from grape-like plastid clusters, giant plastids, or stromules with YFP signals from mitochondria in *atminE1* was observed (**Figures 4A,B**), as was the case for plastid bodies and stromules of WT (**Figure 1E**, middle). Meanwhile, due to the higher surface area of plastids in the mutant, attachment of mitochondria to plastids was more frequently observed in *atminE1* than in WT. Although the bulge structures, which were attached to the plastid bodies or stromules, most closely resembled mitochondria, their CFP signals did not perfectly coincide with mitochondrial YFP signals (**Figure 4C**), indicating that these subplastidic structures and mitochondria were mutually discrete compartments.

At present, we cannot completely exclude the possibility that the occurrence or dynamics of bulge formation might be regulated by mitochondria through their contact with plastids. In the current study, however, we did not find any significant association between plastid structures and mitochondrial morphology/localization. Since the coincidental behavior of stromules and the endoplasmic reticulum has been reported (Schattat et al., 2011a,b), the interaction of plastids and their derived structures, including stromules, with other organelles still deserves further investigation in future studies.

### Genetic Complementation of *atminE1* with *AtMinE1-YFP*

**FIGURE 5:**
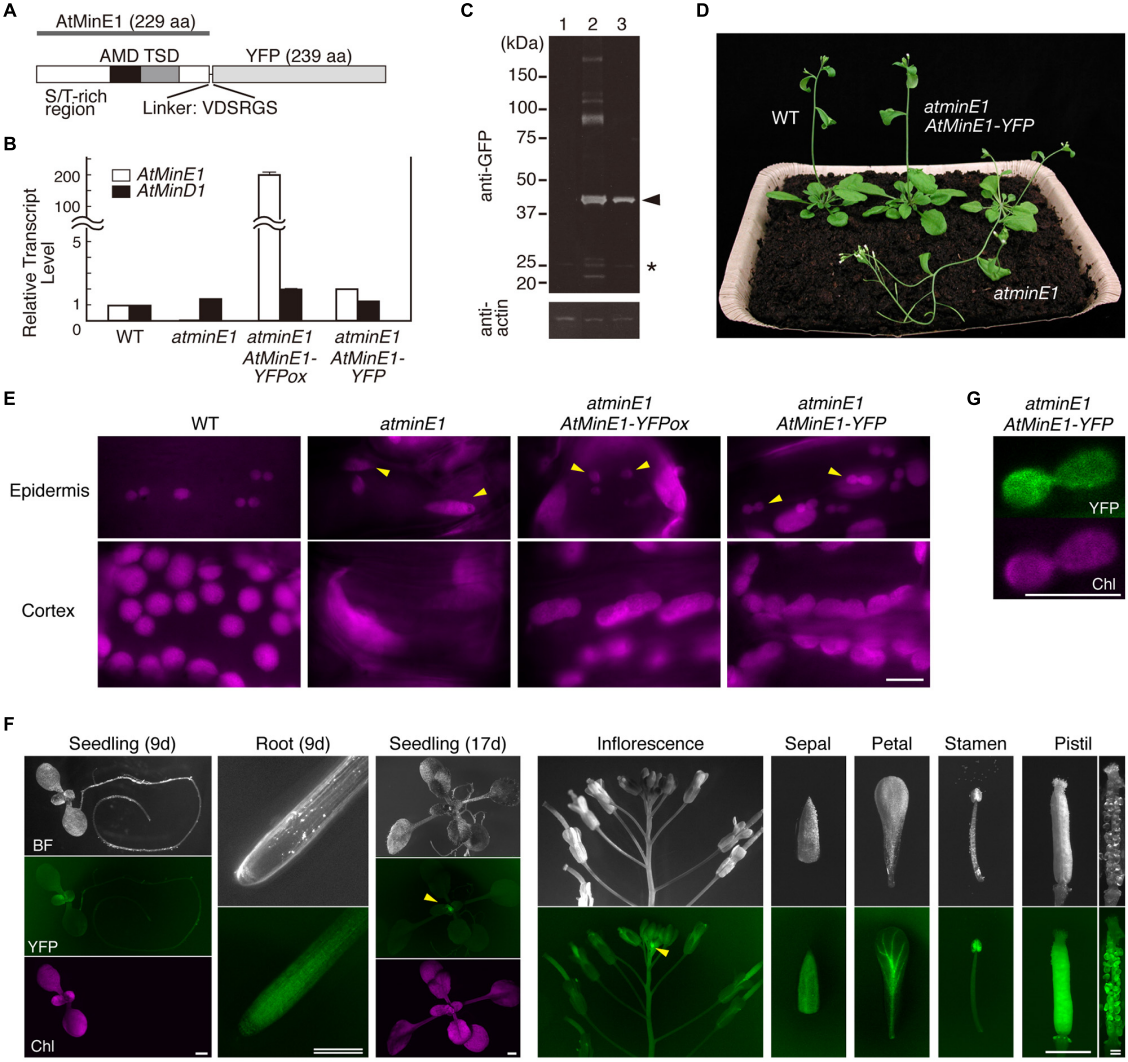
**Expression and function of *AtMinE1-YFP* in *atminE1*. (A)** Domain structure of AtMinE1-YFP. Three regions of AtMinE1, an S/T-rich N-terminal region, an *E. coli* AMD (anti-MinCD domain)-like region, and an *E. coli* TSD (topological specificity domain)-like region, as well as a linker sequence between AtMinE1 and YFP, are indicated. **(B)** Quantitative RT-PCR analysis. RNAs from leaves of WT, *atminE1*, and transgenic *atminE1* with *AtMinE1*p::*AtMinE1-YFP* (an overexpression line and a complemented line) were analyzed. Relative amounts of *AtMinE1* (white bars) and *AtMinD1* (black bars) transcripts compared to *18S rRNA* (WT = 1) are shown. **(C)** Immunoblotting. Proteins from seedlings of WT (lane 1) and transgenic *atminE1* plants (an overexpression line [lane 2] and a complemented line [lane 3]) were analyzed using mouse anti-GFP and anti-actin antibodies. Chemiluminescent signals of AtMinE1-YFP (arrowhead) and non-specific, extra signals (^∗^) are indicated at the right. **(D)** Complementation of plant phenotype of *atminE1* by *AtMinE1-YFP*. One-month-old WT, *atminE1*, and a complemented plant are shown. **(E)** Complementation of plastid morphology of *atminE1* by *AtMinE1-YFP*. Images of chlorophyll autofluorescence from epidermal (top; from 3-week-old seedlings) and cortex (bottom; from 2-week-old seedlings) plastids of WT, *atminE1*, and transgenic *atminE1* plants are shown. Arrowheads represent epidermal plastids. Bar = 10 μm. **(F)** Fluorescence stereomicroscopy. A complemented *atminE1* line at both the vegetative and reproductive stages was observed. Images of bright field (BF), YFP fluorescence (green), and chlorophyll autofluorescence (Chl, magenta) are shown. Arrowheads indicate accumulation of YFP signals at the shoot apices. Bars = 1 mm (single) and 200 μm (double). **(G)** Localization of AtMinE1-YFP signals in leaf epidermal plastids. Fluorescence images of YFP (green) and chlorophyll (magenta) in a leaf epidermal cell of the complemented *atminE1* line are shown. Bar = 5 μm.

To elucidate the relationship between the mutant phenotypes and the function of AtMinE1, it is important to examine whether *AtMinE1* is expressed in leaf epidermal cells. To investigate the expression profile of AtMinE1 *in planta*, we previously performed GUS staining using transgenic *A. thaliana* plants harboring an *AtMinE1*-upstream genomic sequence::*uidA* fusion (Itoh et al., 2001), revealing that GUS activation occurred strongly in the shoot apex and moderately in green tissues and pollen, but not in roots, in most transgenic plants. One exceptional line (Itoh et al., 2001) exhibited GUS staining in whole plants including roots, and we recently considered the possibility that this exceptional transgenic line might represent *AtMinE1* expression in light of a comprehensive *A. thaliana* transcriptome study (Winter et al., 2007) and a study of non-photosynthetic plastids of *atminE1* (Kojo et al., 2009). We constructed an *AtMinE1* promoter::*AtMinE1-YFP* fusion gene (**Figure 5A**) and introduced it into the nuclear genome of *atminE1* via *Agrobacterium*-mediated transformation. The complemented *atminE1* transgenic plants, if obtained, would produce almost WT levels of AtMinE1 fused to a visual reporter subjected to control at the transcriptional, splicing, translational, and post-translational levels.

We obtained 24 T_1_ lines with bialaphos resistance conferred by the T-DNA. Of these, two lines showed complemented phenotypes, 11 showed partially complemented phenotypes, nine showed phenotypes that were defective in plastid division site placement, and two showed no effects, based on observations of epidermal plastids and cortex chloroplasts in leaf petioles (details described below). We chose one division site placement-defective line and one complemented line for further study. Quantitative RT-PCR analysis revealed that the former (hereafter referred to as the “overexpression line”) had more than 200-fold higher levels of *AtMinE1* transcripts (including the innate *AtMinE1* and the introduced *AtMinE1-YFP*) compared to WT, while the latter (hereafter referred to as the “complemented line”) had an *AtMinE1* expression level approximately twice that of WT (**Figure 5B**). No marked differences in *AtMinD1* expression were detected in the plants examined (**Figure 5B**). We examined the expression of AtMinE1-YFP by Western blotting using an anti-GFP antibody (**Figure 5C**). In both transgenic lines, a major band was specifically detected at 46 kDa, which roughly corresponds to the expected size of AtMinE1-YFP (22.4 kDa [predicted mature form of AtMinE1] plus 26.8 kDa [YFP] plus 0.6 kDa [linker sequence]). The intensity of the main 46-kDa band in the overexpression line was 1.7-fold higher than that of the complemented line. Furthermore, several high-molecular-weight proteins (>80 kDa) were expressed in the overexpression line, suggesting that overexpression of AtMinE1-YFP could result in the formation of stable (SDS-resistant) dimers or multimers of AtMinE1-YFP or heteromeric complexes with its interacting proteins. These results indicate that AtMinE1-YFP was synthesized in these lines as intended. The overexpression of AtMinE1-YFP might have been caused by positional effects of transgene insertion on the chromosomes of transgenic plants. At the macro-morphological level, the inflorescence stems of *atminE1* exhibited reduced gravitropism and an early flowering phenotype, flowering 4 days earlier than WT, under our experimental conditions (**Figure 5D**), although plant morphology and reproduction in this mutant are normal (Fujiwara et al., 2008). This gravisensitivity of *atminE1* showed consistency with the reported phenotype of *arc12*, another mutant of *AtMinE1* (Pyke, 1999; Yamamoto et al., 2002; Glynn et al., 2007). Restored gravitropism and flowering time were observed in both the complemented (**Figure 5D**) and overexpression lines (data not shown). We investigated the extent of complementation of the *atminE1* phenotype by observing chlorophyll autofluorescence in leaf petioles (**Figure 5E**). In WT, epidermal plastids were uniformly sized, with a spherical or dumbbell-shaped (dividing) appearance. By contrast, most epidermal plastids of *atminE1* were relatively large and heterogeneously sized. In both the complemented and overexpression lines, the plastid morphology in the petiole epidermis was similar to that of WT, but the plastid size in the latter was weakly heterogeneous. In the leaf petiole cortex, *atminE1* chloroplasts were enlarged with no detectable constriction sites, as described previously (Fujiwara et al., 2008, 2009), while those of the overexpression line were elongated and had multiple constriction sites, indicative of defects in division site placement. The overexpression of AtMinE1-YFP could have disrupted division site placement via the ARC3-mediated mechanism (Zhang et al., 2013). In the petiole cortex of the complemented line, the chloroplast phenotype conferred by the *atminE1* mutation was almost restored to the WT phenotype with respect to their size, morphological uniformity, and the absence of multiple constrictions, although their chloroplasts were only slightly larger than those of WT. Taken together, these results indicate that AtMinE1-YFP plays a role equivalent to that of the native AtMinE1 and exhibits a relationship between gene expression level and chloroplast phenotype as previously demonstrated (Fujiwara et al., 2008). Therefore, the complemented *atminE1* transgenic line is useful for analyzing AtMinE1 expression *in situ*.

We examined this line at the vegetative and reproductive stages by fluorescence stereomicroscopy (**Figure 5F**). YFP fluorescence was detected in all organs examined: cotyledons, leaves, stems, roots, floral organs (sepals, petals, stamens, pistils), and ovules. AtMinE1-YFP was also expressed in pollen grains (data not shown). Importantly, intense YFP signals were detected at the apical regions of both shoots and roots, and the fluorescence intensities gradually declined as the organs or tissues matured. These YFP fluorescence patterns in seedlings and floral organs spatially overlapped with the GUS expression data, although newly observed signals were present in roots and were faint and broadly distributed within each organ. While validating the newly detected *AtMinE1* expression patterns in the complemented line, we observed chloroplasts at the basal regions of the petal epidermis (Supplementary Figure S3). The morphological phenotype of chloroplasts was restored to that of WT with concomitant AtMinE1-YFP expression in the cells. These results indicate that *AtMinE1* is globally expressed in vegetative and reproductive plant organs and is expressed at high levels in young tissues, with maximum expression occurring in the shoot apex.

We further examined the subcellular localization of AtMinE1-YFP in leaf epidermal cells of the complemented plants (**Figure 5G**). YFP signals were exclusively localized to plastids, with diffuse signals present throughout an area of deeply constricted organelles. The morphological phenotype of the plastids was restored to that of WT, with AtMinE1-YFP fluorescence detected within the plastids.

Altogether, these results demonstrate that the role of AtMinE1 is intimately associated with plastid division and morphogenesis in the leaf epidermis, and its deficiency causes abnormal plastid phenotypes, likely as a direct consequence rather than a secondary effect. Moreover, this new information about AtMinE1 expression in *A. thaliana* also suggests that the plastid phenotypes observed in diverse tissues of *atminE1* (Kojo et al., 2009) may be closely associated with the role of AtMinE1 in these tissues.

### Subplastidic Localization of AtFtsZ1-1 and Plastid Constriction in the Leaf Epidermis of the *atminE1* Mutant

**FIGURE 6:**
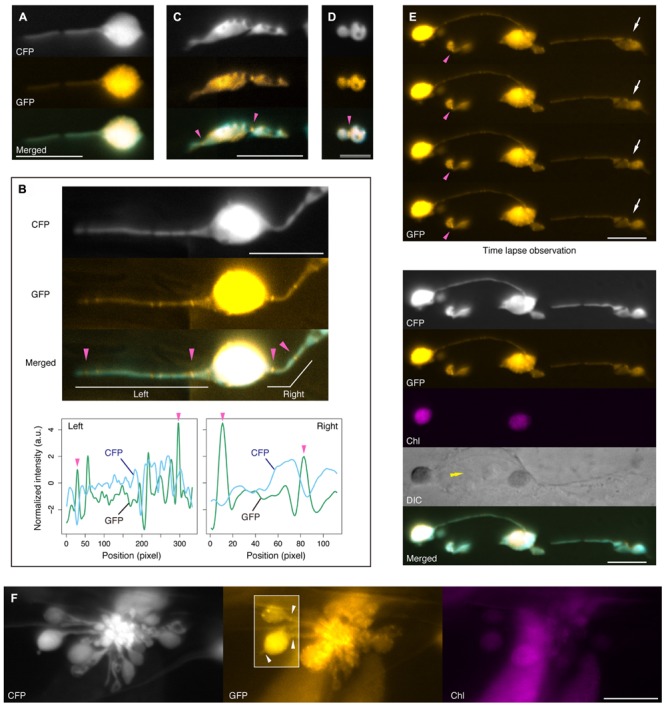
**FtsZ1 localization in stromules and plastid bulges in leaf epidermis of *atminE1*. (A–F)** Dual detection of FtsZ1–GFP and stroma-targeted CFP in epidermal plastids of *atminE1* leaf petioles. Fluorescence images of CFP, GFP (orange-colored), chlorophyll (magenta-colored), and merged (CFP cyan-colored, GFP orange-colored) are shown. In **(B)**, line profiles of normalized CFP and GFP signal intensity in left and right stromules in the image are also presented. Single arrowheads indicate position of FtsZ1 ring placement, while box in **(F)** highlights FtsZ1 ring placement at stromules or between plastid body and plastid vesicle within a grape-like plastid association. Arrows and a double arrowhead in **(E)** indicate a plastid constriction event by time-lapse microscopy and the position of cell nucleus, respectively. Bars = 5 μm **(D)**, and 10 μm (others).

In mesophyll chloroplasts of WT *A. thaliana* plants, AtMinE1 is involved in the formation of the mid-chloroplast FtsZ ring, an early event in chloroplast division (Fujiwara et al., 2008). In fact, in enlarged mesophyll chloroplasts of the *atminE1* mutant, AtFtsZ1-1 (hereafter referred to as FtsZ1) fails to assemble into a mid-chloroplast ring but instead forms various structures such as multiple dots, short filaments, and small, isolated rings (Fujiwara et al., 2009). In this study, we examined the localization of FtsZ1 in the *atminE1* leaf epidermis using a transgenic line in which the FtsZ1–GFP fusion was expressed under its own promoter (Fujiwara et al., 2008). Previously, we observed multiple FtsZ1 rings in mini-sized plastid bodies in the *atminE1* leaf epidermis (Fujiwara et al., 2009). Here, by simultaneously utilizing FtsZ1–GFP and stroma-targeted CFP, we further monitored FtsZ1 localization (with great precision) within stromules and bulges in addition to plastid bodies (**Figure 6**).

In many cases, the signal intensity of FtsZ1–GFP was weak. Like stroma-targeted CFP, FtsZ1–GFP appeared to accumulate much more strongly in the plastid bodies than in stromules, probably reflecting differences in stroma volume (**Figure 6A**). In those plastids, we did not find any characteristic structures of FtsZ1 in their bodies and stromules, except for unfixed dots, which were occasionally observed. While we detected apparent disconnection and constriction of the stromal CFP signal within a single stromule, no particular FtsZ structure was associated with these disconnected or constricted parts of stromules (**Figure 6A**). In certain cases, however, FtsZ1 rings were observed in stromules (**Figure 6B**). As chloroplast division progresses, the appearance of chloroplast FtsZ rings under a fluorescence microscope changes from two dots (at both sides of the isthmus) or a filament (traversing the isthmus) to a single patch (a putative equivalent of the compacted ring; Vitha et al., 2001; Kuroiwa et al., 2002). Since stromules are very thin, FtsZ rings in stromules are considered to be equivalent to those at a late or final stage of normal chloroplast division. Therefore, it was not easy to discriminate between FtsZ1 rings and non-ring structures in stromules, and thus we relied on the following criteria for discrimination: when in focus in the top focal plane of the FtsZ1 ring, the diffusion signal of FtsZ1–GFP dropped at the flanking regions, while the stromal CFP signal dropped at the site of FtsZ1 ring formation, both due to the presence of a ring-associated isthmus. For reference, the FtsZ1 ring in wild-type, dividing plastids (**Figure 1E**, right) obviously met these criteria. In light of these criteria, in the plastid shown in **Figure 6B**, at least four FtsZ1 rings were recognized (magenta arrowheads) in the two stromules emanating from the single body. As shown in **Figures 6A,B**, FtsZ1 rings were not necessarily associated with the constricted/disconnected parts of stromules. However, in some cases, unfixed dots of FtsZ1–GFP were observed within the diffusion signal. Most of these FtsZ1 dots could be distinguished from FtsZ1 rings (**Figure 6B**) based on the above criteria. In the *atminE1* leaf epidermis, we also observed a putative FtsZ1 ring at one or more constriction site(s) of the plastid main bodies (**Figures 6C,D**). In such plastids, FtsZ1–GFP signals were concentrated either in a short filament traversing the shallow constriction or in a spot at the deep constriction (**Figures 6C,D**, magenta arrowheads) in addition to the stromal diffusion signal. Since epidermal plastids in *atminE1* are highly irregular and pleomorphic, it was previously difficult to judge whether such a plastid was undergoing fission merely based on its shape. It is noteworthy that the use of FtsZ1–GFP in combination with stromal CFP enabled us to identify epidermal plastids undergoing fission (**Figure 6C**, for example).

Based on the above observations, together with our previous findings (Fujiwara et al., 2009), we postulate that, even without AtMinE1, (i) FtsZ1 could assemble into a ring in non-swollen plastid bodies, stromules and bulges, and (ii) the formation of the FtsZ1 ring could lead to constriction and, ultimately, membrane fission of those plastid compartments. While we have provided evidence for postulate (i), postulate (ii) has yet to be verified experimentally. To address this issue, we performed time-lapse fluorescence imaging of FtsZ1–GFP according to the method of Fujiwara et al. (2009), demonstrating that plastid bodies were capable of isthmus formation and constriction without AtMinE1 (**Figure 6E**). Intriguingly, as exemplified by the plastid shown in **Figure 6E**, the constricting isthmus of plastid bodies often appeared to lack a clear FtsZ1 ring. In leaf epidermal plastids, FtsZ1 ring formation may not be a prerequisite for the constriction of plastid bodies.

Within grape-like plastid aggregations, putative FtsZ1 rings were observed in the narrow, stromule-like regions of bulges, whereas the vast majority of FtsZ1–GFP fluorescence was detected as a diffuse signal within the plastid stroma (**Figure 6F**; see also Supplementary Figure S4). The apparent aggregations of plastids that occasionally occurred in the *atminE1* leaf epidermis (**Figures 2E, 3H, 4A** and **6F**, Supplementary Figure S2C) may have resulted from the failure of the stroma-containing bulges (initially in the form of tubes, lobes, or vesicles) to separate; the FtsZ1 rings in the plastid aggregations (**Figure 6F**) might represent suspended plastid fission. In other words, the “plastid aggregate” might, in fact, represent a single or a few plastid(s), inside which a number of bulges formed and grew, rather than a product of massive connection or aggregation of independent plastid bodies and bulges.

Based on our detection of plastid FtsZ1 rings (**Figures 6B–D,F**) and time-lapse imaging of plastids (**Figure 6E**), we conclude the following: (1) In the *atminE1* leaf epidermis, the formation of FtsZ1 rings can occur both within non-giant plastid bodies and within plastid bulges and stromules. (2) Deep plastid constrictions with FtsZ1 rings can be generated in two ways, either by constriction of FtsZ1 rings in the plastid bodies or by sustained presence of FtsZ1 rings in the bulges/stromules in conjunction with the growth of both sides of the ring. (3) Because of depletion of AtMinE1, the FtsZ1 rings at deep constrictions can inefficiently (or scarcely) mediate the scission of plastid envelope membranes and, as a result, may help stabilize the constrictions by counteracting the swelling and deformation of plastid bodies, bulges, or stromules.

### MinE-independent FtsZ1 Ring Formation and Plastid Constriction: Hidden Diversity of Plastid Division/Constriction Patterns in Leaf Tissues

Epidermal plastids replicate by FtsZ1 ring-associated binary fission, in the same manner as mesophyll chloroplasts (**Figure 1E**, right). Despite this, the effects of a mutation in *AtMinE1* on plastid division and morphology differed markedly, even in the same leaf, depending on tissue type. Our present data provide insight into the process by which epidermal plastids become pleomorphic. First, our observations indicate that FtsZ1 ring-mediated membrane constriction occurs in stromules, bulges, and small plastid bodies, but not in giant plastids, in the absence of AtMinE1, although it remains unknown whether the FtsZ1 ring is actually capable of stromule fission. The above data support our previously proposed hypothesis (Fujiwara et al., 2008, 2009) that excessive expansion of plastids may inhibit FtsZ-based constriction, while plastids with a small diameter and stromules may be capable of efficient constriction. Second, this study suggests that FtsZ1 can assemble into a ring and produce a membrane constriction, even without AtMinE1 (**Figure 6B**), but it appears unable, or scarcely able, to complete membrane fission in the (almost) complete absence of AtMinE1. This finding implies that there is a novel, MinE-independent mode of plastid division/constriction, which might be specific to the epidermal or non-green tissue. Such FtsZ1 rings at the plastid constrictions may play an envelope-tightening or holding role in the AtMinE1-deficient epidermis. It is possible that the MinE-independent mode is also present in stromules in the epidermis of WT plants but is hidden, perhaps due to the relatively low number and short length of the stromules in WT or to the completion of “stromule fission” by the functional FtsZ1 ring, which would produce difficult-to-identify plastid-derived structures. In this context, it is interesting that detachment of a vesicle-like plastid from the tip of the stromule, a process known as “tip-shedding”, was previously observed in trichome cells of tomato (*Solanum lycopersicum*) using video microscopy with differential interference contrast optics (Gunning, 2005).

Assuming the above hypotheses are correct, we can explain the terminal phenotype of plastid morphology in the *atminE1* epidermis as the following “stromule-derived subcompartments” model: (Step 1) stromules and bulges emanating from giant plastids undergo FtsZ-based constriction to produce even smaller subcompartments, while the giant plastid bodies themselves cannot constrict due to their large diameter; (Step 2) these smaller subcompartments undergo further rounds of constriction, producing still more subcompartments; (Step 3) these subcompartments may continuously grow wider or longer whereas the pre-existing constrictions largely remain unchanged due to the presence of static FtsZ1 rings; and (Step 4) the above steps are repeated in epidermal cells, giving rise to a population of plastids with rapidly increasing shape heterogeneity. This model would also explain how the “grape-like” plastid clusters (**Figures 2E, 3H, 4A and 6F**, Supplementary Figure S2C) could be generated from a single or a few plastids, while such images are usually interpreted as the aggregation and connection of several independent plastids (the “connected plastids” model). As another example, the plastid image in **Figure 2A** may have been interpreted as multiple plastids connected by stromules according to the conventional viewpoint (the “connected plastids” model). However, based on the new viewpoint, this image can be interpreted as a single giant plastid whose stromules have grown into subplastid bodies or bulges (the “stromule-derived subcompartments” model). Localization of FtsZ1 rings at the constriction sites of plastid bulges and stromules (**Figure 6**) also supports the present “stromule-derived subcompartments” model rather than the conventional “connected plastids” model.

## Conclusion

In previous studies, terminal phenotypes of various chloroplast division mutants were interpreted based on the established framework of mesophyll chloroplast division. Specifically, plastid enlargement has been regarded as an indicator that plastids cannot divide, and heterogeneity of plastid size was thought to indicate that plastid division (and associated formation of the FtsZ ring) had occurred at abnormal (i.e., non-central and random) site(s). However, the terminal phenotype of plastids in the *atminE1* epidermis could not be fully explained by this model. Our results suggest that the well-studied control of mesophyll chloroplast division is not necessarily true for plastid replication in other leaf tissues. Our data also emphasize the need to establish a framework of non-mesophyll plastid division, which would undoubtedly be important for elucidating the development and differentiation of plastids.

## Author Contributions

MF, RI designed the study, interpreted the data, and wrote the paper. MF, YK, SS, RI performed the experiments. MF, KK conducted the image analysis. TA, MF, RI contributed reagents/materials/analysis tools.

## Conflict of Interest Statement

The authors declare that the research was conducted in the absence of any commercial or financial relationships that could be construed as a potential conflict of interest.
