# Supplementary material for: The Arabidopsis minE mutation causes new plastid and FtsZ1 localization phenotypes in the leaf epidermis
Source: Front Plant Sci. 2015 Oct 6;6:823. doi: 10.3389/fpls.2015.00823 (PMC4593956; doi:10.3389/fpls.2015.00823)
Supplement: Supplementary file 1 [file Data_Sheet_1.PDF]

Supplementary Material

The *Arabidopsis minE* mutation causes new plastid and FtsZ1 localization phenotypes in the leaf epidermis

Makoto T. Fujiwara, Kei H. Kojo, Yusuke Kazama, Shun Sasaki, Tomoko Abe and Ryuuichi D. Itoh*

*** Correspondence:** Ryuuichi D. Itoh: ryuitoh@sci.u-ryukyu.ac.jp

1. Supplementary Figures

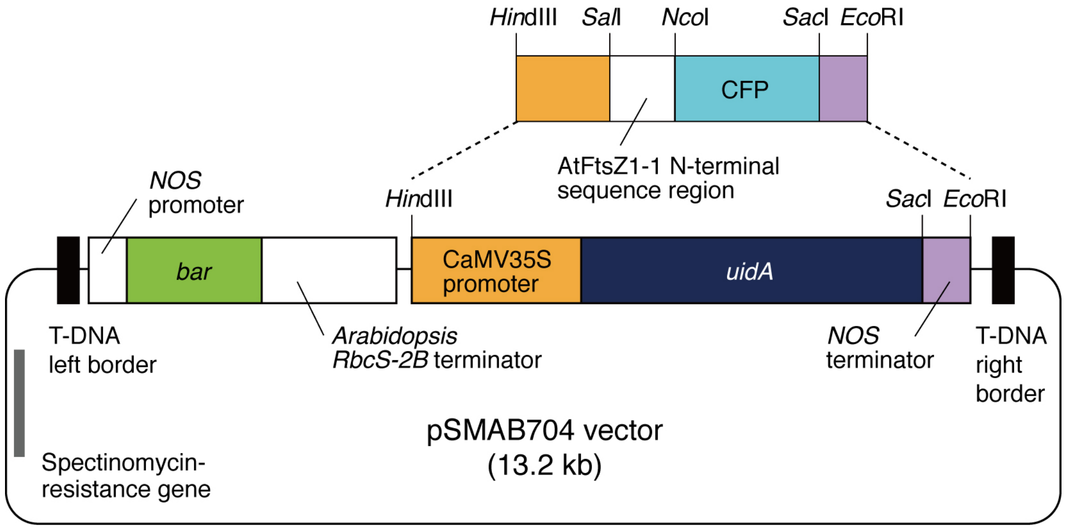

Supplementary Figure 1. Construction of a transformation vector expressing stroma-targeted CFP in *Arabidopsis*. The original vector pSMAB704 was modified to carry a CaMV35S promoter::$TP_{FtsZ1\text{-}1}$-*CFP*::*NOS* terminator gene cassette. Representative genes and restriction sites are indicated on the map. See also Materials and Methods.

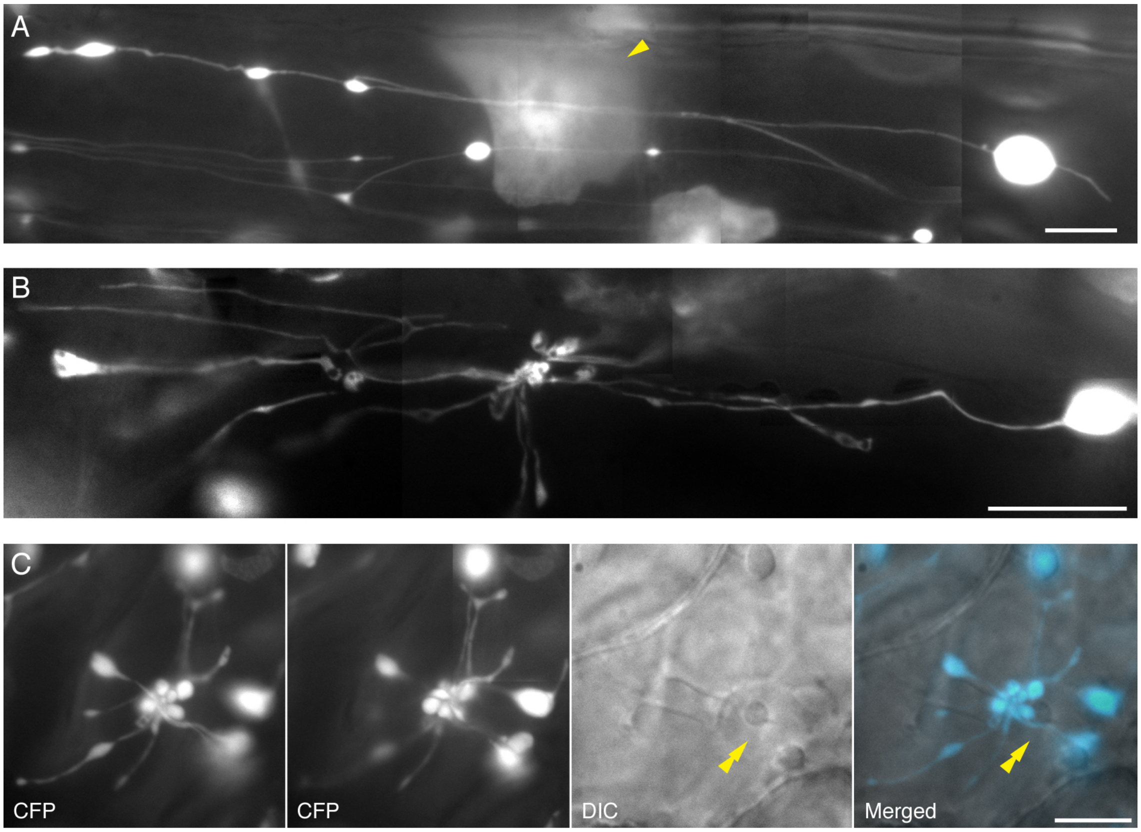

Supplementary Figure 2. Plastid morphology in leaf epidermis of *atminE1*. (A–C) Images of CFP-labeled plastids in leaf petiole epidermis of 2- or 3-week-old *atminE1* seedlings. In **(C)**, two CFP images taken at different foci, DIC, and merged (CFP cyan-colored) images are shown. Single and double arrowheads indicate a cortex chloroplast and nucleus, respectively. Bars = 10 μm.

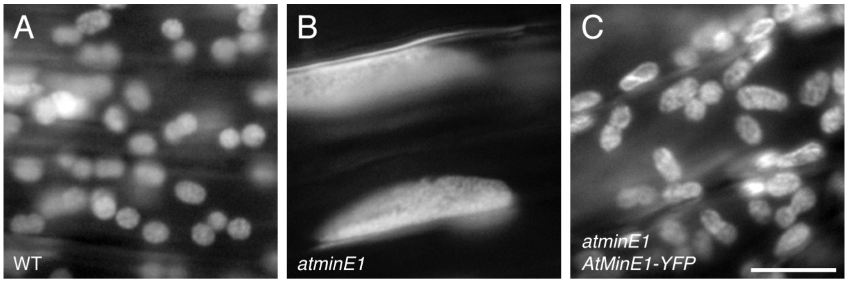

Supplementary Figure 3. Complementation of aberrant plastid morphology of *atminE1* by *AtMinE1-YFP*. Images of chlorophyll autofluorescence from epidermal plastids at the petal base of WT, *atminE1*, and a complemented transgenic *atminE1* plant are shown. Bar = 10 μm.

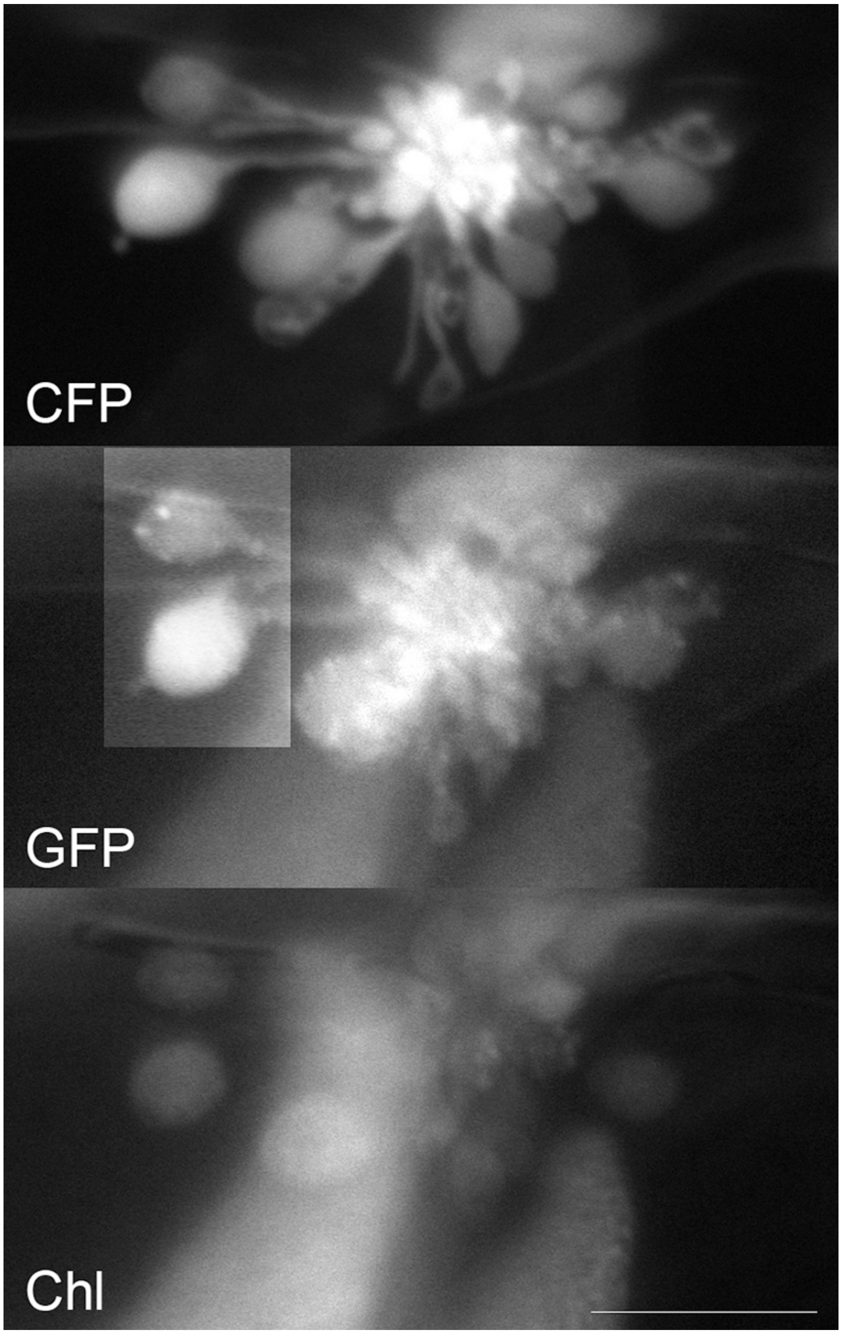

Supplementary Figure 4. Grape-like plastid cluster in leaf epidermis of *atminE1*. A magnified version of Fig. 6F is presented to demonstrate the chlorophyll autofluorescence image (Chl, bottom panel) more clearly. Fluorescence images of stroma-targeted CFP (CFP, top panel) and FtsZ1-GFP (GFP, middle panel) are also shown. Box in the GFP panel highlights a region containing putative FtsZ1 rings at stromules or between plastid body and plastid vesicle. Bar = 10 µm.
